# Supplementary material for: Spatial Variability in Streambed Microbial Community Structure across Two Watersheds
Source: Microbiol Spectr. 2021 Dec 15;9(3):e01972-21. doi: 10.1128/Spectrum.01972-21 (PMC8672884; doi:10.1128/Spectrum.01972-21)
Supplement: SUPPLEMENTAL FILE 1 — Supplemental material. Download SPECTRUM01972-21_Supp_1_seq2.pdf, PDF file, 0.3 MB [file spectrum01972-21_supp_1_seq2.pdf]

**Supplemental Material:**

# Spatial Variability in Streambed Microbial Community Structure Across Two Watersheds

**Philips O. Akinwole[a,c]*, Jinjun Kan[b], Louis A. Kaplan[b] and Robert H. Findlay[c]**

[a]Biology Department, DePauw University, Greencastle, IN 46135 USA

[b]Stroud Water Research Center, 970 Spencer Road, Avondale, PA 19311 USA

[c]Department of Biological Sciences, University of Alabama, Tuscaloosa, AL 35475 USA

* Corresponding author:

Biology Department, DePauw University, Greencastle, IN 46135

philipsakinwole@depauw.edu

**Supplemental Material**

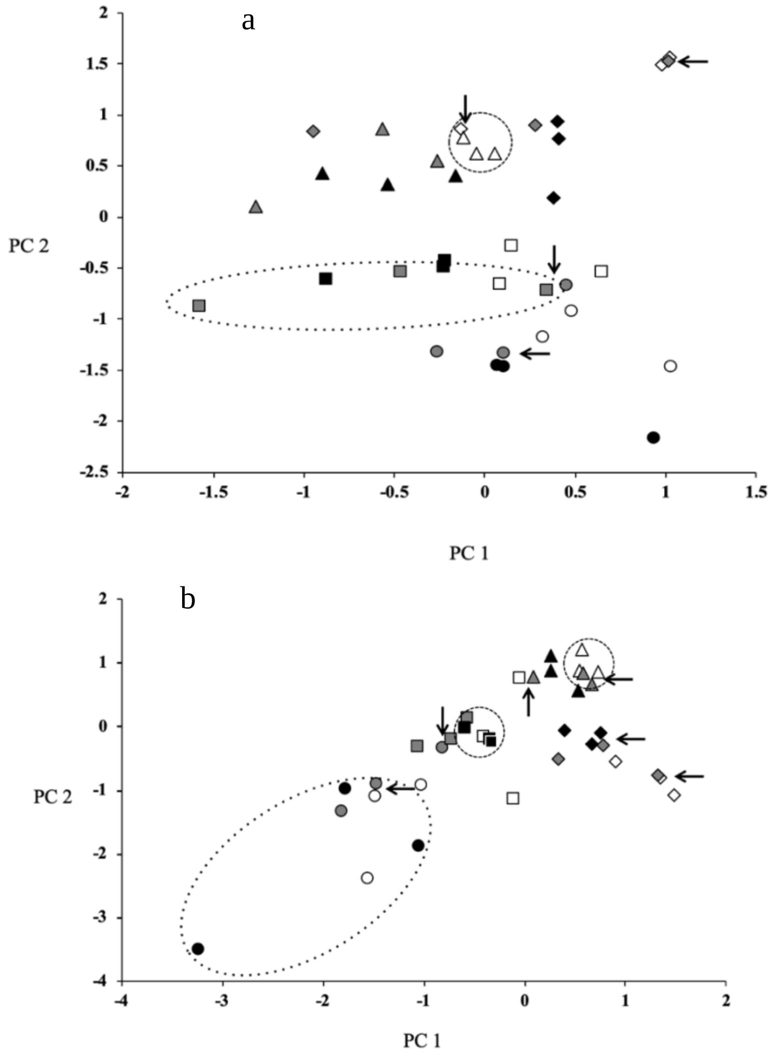


**FIG S1.** a) Comparison of microbial community structure among individual cores from four selected streams: Diamonds – LSB; triangles - WCS; squares - PBR; circles - BBR. Symbol color denotes station, with white indicating upper reach, gray middle reach and black lower reach, respectively. The dashed circle indicates the station with lowest among adjacent core variation while the dotted ellipse indicates the station with the highest among adjacent core variation. The horizontal arrow points to similar composition among cores (different colors) within the same stream reach (same shape), while vertical arrows point to similar composition between cores (of any color) in different reaches (different shapes). b) Comparison of bacterial community structure among individual cores from four selected streams. Samples and their relationships are indicated as above with the addition of an upward pointing arrow indicating two samples from the two different watersheds with very similar community structure.

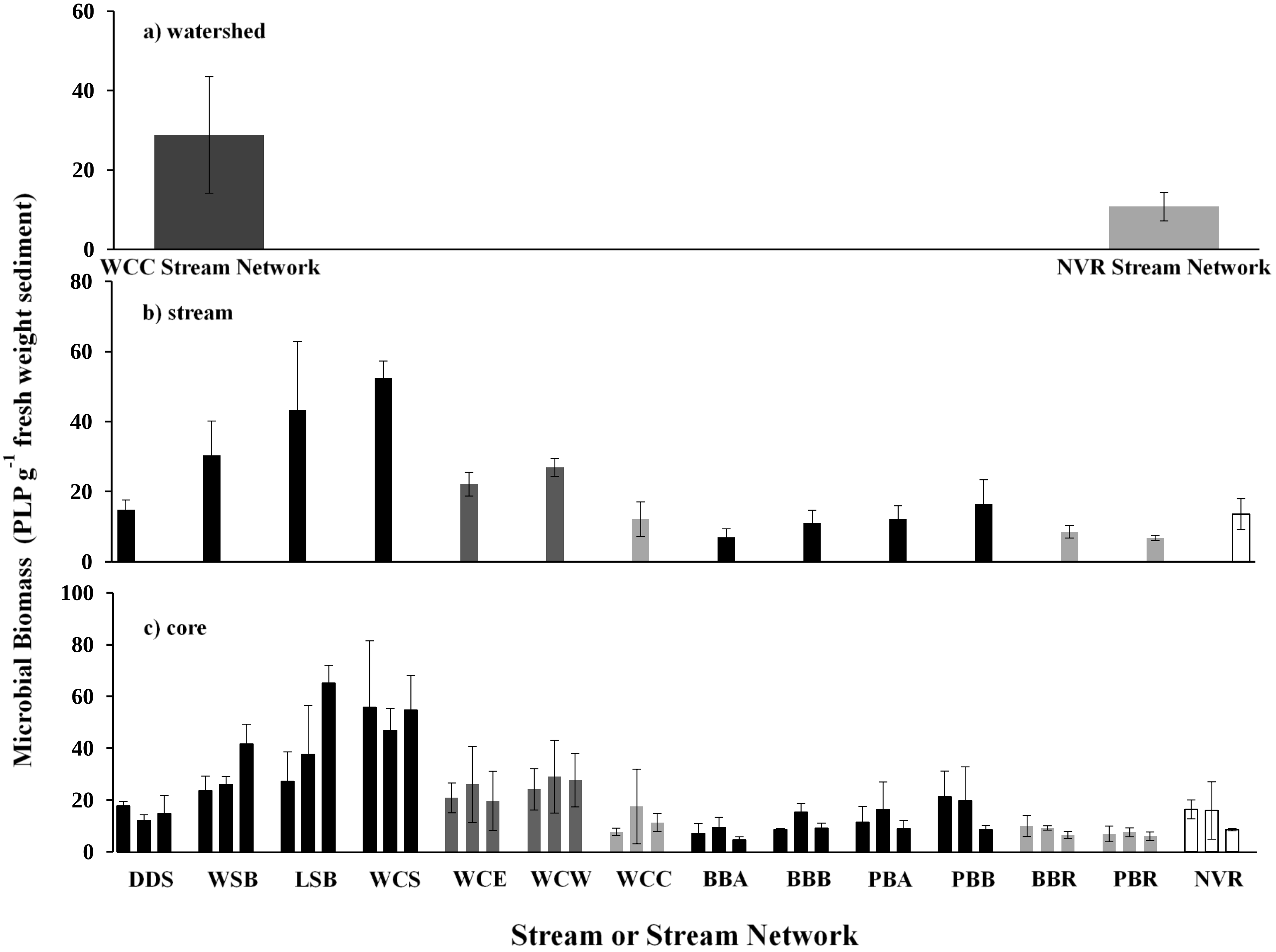

a) watershed
WCC Stream Network
NVR Stream Network
b) stream
c) core
Microbial Biomass (PLP g-1 fresh weight sediment)
DDS
WSB
LSB
WCS
WCE
WCW
WCC
BBA
BBB
PBA
PBB
BBR
PBR
NVR
Stream or Stream Network

**FIG S2.** Microbial biomass (mean ± SD) of White Clay Creek and Neversink watershed sediments at three spatial scales: a; watershed, b; stream and c; station. Stream order (or average order for watershed values) are indicated as: black = 1st order, dark gray = 2nd order, light gray = 3rd order, open = 5th order.

**Table S1**. Microbial biomass and composition, water chemistry and sediment organic content of White Clay Creek and Neversink watersheds.

| Watershed & stream | Biomass/PLP (nmol $g^{-1}$ fresh wet wt)[a] | Bacterial abundance ($g^{-1}$ fww)[b] | % Eukaryotic/ prokaryotic[c] | Cond (µS/cm) | Temp ($^0$C) | % C | % N | C:N ratio[d] |
|---|---|---|---|---|---|---|---|---|
| White Clay Creek | | | | | | | | |
| DDS | 14.80 ±2.81 | 5.67 x $10^8$ | 4/96 | 186.0 | ND | 0.33 ±0.01 | 0.02 ±0.55 | 14.14 ±3.18 |
| WSB | 30.34 ±9.81 | 8.24 x $10^8$ | 31/69 | 225.0 | ND | 0.58 ±0.02 | 0.04 ±0.54 | 12.98 ±2.53 |
| LSB | 43.29 ±19.60 | 1.56 x $10^9$ | 9/91 | 373.6 | ND | 2.96 ±0.37 | 0.31 ±0.51 | 12.77 ±5.12 |
| WCS | 52.41 ±4.87 | 1.68 x $10^9$ | 20/80 | 303.3 | ND | 3.36 ±0.15 | 0.21 ±0.34 | 16.45 ±2.09 |
| WCE | 22.13 ±3.38 | 8.26 x $10^8$ | 6/94 | 189.9 | ND | 1.15 ±0.04 | 0.07 ±0.37 | 16.27 ±2.75 |
| WCW | 26.89 ±2.52 | 1.01 x $10^9$ | 6/94 | 259.7 | 16.2 | 1.73 ±0.11 | 0.11 ±0.37 | 16.59 ±2.71 |
| WCC | 12.12 ±4.95 | 4.25 x $10^8$ | 11/89 | 232.7 | 16.1 | 0.57 ±0.04 | 0.06 ±5.03 | 9.08 ±2.96 |
| Neversink | | | | | | | | |
| BBA | 6.99 ±2.39 | 2.27 x $10^8$ | 19/81 | 35.1 | 15.4 | 0.36 ±0.01 | 0.03 ±1.08 | 11.15 ±2.30 |
| BBB | 10.95 ±3.74 | 3.34 x $10^8$ | 23/77 | 32.4 | 14.8 | 0.34 ±0.01 | 0.03 ±0.55 | 10.25 ±3.15 |
| PBA | 12.18 ±3.78 | 3.92 x $10^8$ | 18/82 | 25.6 | 16.2 | 0.72 ±0.04 | 0.05 ±0.35 | 12.99 ±3.10 |
| PBB | 16.42 ±6.96 | 5.73 x $10^8$ | 12/88 | 18.8 | 15.5 | 1.18 ±0.11 | 0.08 ±0.38 | 11.06 ±3.30 |
| BBR | 8.54 ±1.79 | 2.87 x $10^8$ | 16/84 | 20.8 | 16.4 | 0.18 ±0.00 | 0.03 ±0.30 | 5.62 ±2.38 |
| PBR | 6.77 ±0.75 | 2.03 x $10^8$ | 25/75 | 24.5 | 16 | 0.16 ±0.01 | 0.02 ±0.88 | 6.18 ±3.76 |
| NVR | 13.58 ±4.41 | 2.95 x $10^8$ | 42/58 | 33.5 | 15.3 | 0.20 ±0.01 | 0.03 ±0.71 | 7.69 ±1.05 |

[a] Mean ± standard deviation (n = 9).

[b] Calculated from PLP x % prokaryotic (expressed as decimal fraction) and a conversion factor of 100 nmol PLP = 4 x $10^9$ cells

[c] Percentage that microeukaryotic contributes of total microbial biomass, calculated from PLFA profiles (n = 9).

[d] Percent that C and N contribute to total sediment elementary atoms (n = 9)

ND= measurements were not taken
